# Issue Information

**DOI:** 10.1002/mgg3.51

**Published:** 2013-11-11

## Aims and Scope

*Molecular Genetics & Genomic Medicine* is a peer reviewed journal for rapid dissemination of high-quality research related to the dynamically developing areas of human, molecular and medical genetics. The journal publishes original research articles covering novel fi ndings in phenotypic, molecular, biological, and genomic aspects of genomic variation, inherited disorders and birth defects. The broad publishing spectrum of *Molecular Genetics & Genomic Medicine* includes rare and common disorders from diagnosis to treatment. Examples of appropriate articles include reports of novel disease genes, functional studies of genetic variants, in-depth genotype-phenotype studies, genomic analysis of inherited disorders, molecular diagnostic methods, medical bioinformatics, ethical, legal, and social implications (ELSI), and novel approaches to clinical diagnosis. We anticipate that *Molecular Genetics & Genomic Medicine* will provide a high quality scientifi c home for next generation sequencing studies of rare and common disorders, which will make novel fi ndings in this fascinating area easily and rapidly accessible to the scientific community. This will serve as the basis for translating next generation sequencing studies into individualized diagnostics and therapeutics, for day-to-day medical care.

*Molecular Genetics & Genomic Medicine* publishes original research articles, reviews, and research methods papers, along with invited editorials and commentaries. Original research papers must report well-conducted research with conclusions supported by the data presented.

*Molecular Genetics & Genomic Medicine* is a Wiley Open Access journal, one of a series of peer reviewed titles publishing quality research with speed and effi ciency. For further information visit the Wiley Open Access website.

## Open Access and Copyright

All articles published by *Molecular Genetics & Genomic Medicine* are fully open access: immediately freely available to read, download and share. All *Molecular Genetics & Genomic Medicine* articles are published under the terms of the Creative Commons Attribution License which permits use, distribution and reproduction in any medium, provided the original work is properly cited.

Copyright on any research article in a journal published by *Molecular Genetics & Genomic Medicine* is retained by the author(s). Authors grant Wiley a license to publish the article and identify itself as the original publisher. Authors also grant any third party the right to use the article freely as long as its integrity is maintained and its original authors, citation details and publisher are identifi ed. Further information about open access license and copyright can be found here.

## Purchasing Print Reprints

As this is an open access journal, you have free, unlimited access to your article online. However, if you wish to obtain printed reprints, these may be ordered online: https://caesar.sheridan.com/reprints/redir.php?pub=10089&acro=MGG3

## Disclaimer

The Publisher and Editors cannot be held responsible for errors or any consequences arising from the use of information contained in this journal; the views and opinions expressed do not necessarily refl ect those of the Publisher and Editors, neither does the publication of advertisements constitute any endorsements by the Publisher and Editors of the products advertised. Wiley Open Access articles posted to repositories or websites are without warranty from Wiley of any kind, either express or implied, including, but not limited to, warranties of merchantability, fitness for a particular purpose, or non-infringement. To the fullest extent permitted by law Wiley disclaims all liability for any loss or damage arising out of, or in connection with, the use of or inability to use the content.

